# Trousseau’s Opinion of Tracheotomy

**Published:** 1858-12

**Authors:** 


### EXTRACTS.

[*From the Cincinnati Lancet and Observer.*]

### TROUSSEAU'S OPINION OF TRACHEOTOMY.

Tracheotomy, well performed and badly treated, is invariably fatal; but badly performed and well treated, it succeeds in a third of the cases. The wound ought to be defended by plaster, in order to prevent its edges being injured by the borders of the canula. A woolen cravat ought to be passed three or four times loosely around the neck, so as to absorb the heat and moisture requisite for the air entering the lungs; otherwise, the mucus in the trachea dries up too rapidly, and so inflammation of it is excited. The edges of the wound ought to be cauterized, to prevent their taking on a diphtheritic covering; rub the caustic in well, the day after the operation at latest. Children operated on must be made to eat; insist on this. Despite the fever, prescribe milk, eggs, chocolate, cream, etc., as deglutition is very difficult with children; the best thing is to remove the canula as early as possible. In the angina of the diphtheritic, the canula may be removed on the sixth to the ninth day; but sometimes it must be left for months or years. Blisters must never be used in diphtherite: they only add another complication.—*Med. Times and Gazette.*

